# Extrinsic Calibration of Multiple 3D LiDAR Sensors by the Use of Planar Objects

**DOI:** 10.3390/s22197234

**Published:** 2022-09-23

**Authors:** Hyunsuk Lee, Woojin Chung

**Affiliations:** Department of Mechanical Engineering, Korea University, Seoul 02841, Korea

**Keywords:** extrinsic calibration, multiple LiDAR sensors, plane-based registration, non-linear optimization

## Abstract

Three-dimensional light detection and ranging (LiDAR) sensors have received much attention in the field of autonomous navigation owing to their accurate, robust, and rich geometric information. Autonomous vehicles are typically equipped with multiple 3D LiDARs because there are many commercially available low-cost 3D LiDARs. Extrinsic calibration of multiple LiDAR sensors is essential in order to obtain consistent geometric information. This paper presents a systematic procedure for the extrinsic calibration of multiple 3D LiDAR sensors using plane objects. At least three independent planes are required within the common field of view of the LiDAR sensors. The planes satisfying the condition can easily be found on objects such as the ground, walls, or columns in indoor and outdoor environments. Therefore, the proposed method does not require environmental modifications such as using artificial calibration objects. Multiple LiDARs typically have different viewpoints to reduce blind spots. This situation increases the difficulty of the extrinsic calibration using conventional registration algorithms. We suggest a plane registration method for cases in which correspondences are not known. The entire calibration process can easily be automated using the proposed registration technique. The presented experimental results clearly show that the proposed method generates more accurate extrinsic parameters than conventional point cloud registration methods.

## 1. Introduction

Autonomous navigation technology has been applied to robots and vehicles. Furthermore, commercialization of autonomous navigation systems operating in urban environments has been accelerating in recent years. Consequently, the perception of environment in autonomous systems is becoming increasingly crucial for system and user safety. Therefore, many navigation systems are equipped with a sensor system consisting of multiple sensors of the same type or sensors with different modalities.

Three-dimensional light detection and ranging (LiDAR) sensors provide accurate range measurements and abundant geometric information. A wide range of products for 3D LiDAR sensors has been developed in recent years, placing 3D LiDAR sensors among the most preferred in the field of autonomous navigation. Although the 3D LiDAR sensors that have high vertical resolution and a wide vertical field of view (e.g., Velodyne HDL64) provide a dense point cloud, it is not easy to apply them to commercial autonomous navigation systems because of their exorbitant prices. In this case, the 3D LiDAR sensors that have low vertical resolution (e.g., Velodyne VLP-16) can act as substitutes because the prices of the vertically low-resolution LiDAR sensors are relatively lower than those of the high vertical resolution 3D LiDAR sensors, such as HDL-64. However, they have a narrow vertical field of view. It is essential for an autonomous navigation system to measure a wider range of the environment while lowering the cost of the entire system. Furthermore, there is a need for a sensor system that reduces blind spots. Consequently, multiple 3D LiDAR sensors are often applied to autonomous navigation systems. Because the LiDAR sensors are typically mounted on a robotic or vehicular platform at different locations and directions, the transformation between the sensors should be well defined. From this point of view, extrinsic calibration to estimate the relative position and rotation between the sensors should precede the operation of the autonomous navigation system.

The importance of extrinsic calibration is evident for the following reasons: First, when commercializing autonomous navigation systems equipped with multiple sensors, large numbers of systems need to be calibrated in batches. Second, for research platforms, the poses of the sensors are often adjusted to find a suitable configuration. Furthermore, sensors are often attached and detached while inspecting the platform or reconfiguring the sensor systems. Therefore, the extrinsic calibration is not a one-off operation for the systems. Consequently, an easily applicable and accurate extrinsic calibration method is required.

Regarding environmental perception, it is efficient for autonomous navigation systems to cover a wide ranging area with few sensors where possible, especially when using multiple sensors with the same modality. Therefore, multiple LiDAR sensors are forced to install the viewpoints of the sensors quite differently so that the sensor systems have a wide field of view. Consequently, the overlapping regions between the sensors are reduced. However, for extrinsic calibration, the reduced overlapping regions make it difficult to determine the extrinsic parameters between sensors. For this reason, point cloud registration algorithms [1,2,3,4,5] may fail in sensor configurations with different points of view.

Calibration methods using artificial calibration objects require processes for making the objects and for placing them in the target environments [6,7,8]. To obtain a robust estimation of extrinsic parameters, the calibration objects should be large enough, or they should be sufficient in number. However, artificial calibration objects are generally small, meaning that the measurement points of the objects are only a fraction of the large number of point clouds that LiDAR sensors provide. Consequently, the calibration results are sensitive to sensor noise. Furthermore, it is sometimes necessary to set up a region of interest without automatically detecting the object.

There are several studies on motion-based extrinsic calibration. These methods are based on hand-eye calibration using structure from motion. The extrinsic parameter is calibrated using the motions obtained by each sensor. Because detection of mutual information is not required, it is usually used for the sensors with different modalities, such as LiDAR-Camera-IMU [9] or LiDAR-Camera [10,11]. However, the motion-based techniques require the exact trajectories of the vehicle. Although the trajectory can be obtained via the inertial navigation system (INS) or visual odometry, it is difficult to estimate the exact trajectory of the system. Moreover, the accuracy of the trajectory estimation is affected by the noise of the sensor. Thus, the trajectory estimation errors may result in estimation errors on extrinsic parameters.

In this study, we propose a method for extrinsic calibration of multiple 3D LiDAR sensors. The contributions of this paper are threefold. First, the proposed method uses plane objects in surrounding environments as calibration objects. Specifically, at least three planes whose normal vectors span $R^{3}$ are required. The planes satisfying the condition can easily be found in nearby structures (e.g., the ground, walls, or columns). Therefore, the proposed method does not require environmental modifications such as placing artificial calibration objects. Secondly, we suggest a plane registration method for matching the plane objects detected by each sensor. For sensors with different viewpoints, there are only small overlapping regions. This situation increases the difficulty of assigning plane correspondences. The proposed registration technique can easily automate the entire calibration process without human intervention. Thirdly, we consider the uncertainty of the plane features in the optimization process. Noisy planes may exist among the detected plane features. The reliability of the calibration results can be increased by lowering the weight of the noisy plane.

## 2. Related Work

Several studies have addressed the extrinsic calibration of one or more LiDAR sensors. One possible approach is matching geometrically distinctive features. Choi et al. [12] proposed a method for estimating the relative pose between two 2D LiDAR sensors. A set of planes were used as the calibration object, and the coplanarity and orthogonality of the scan points were considered to determine the extrinsic parameters. In [13], a method for extrinsic calibration of a set of 2D LiDAR sensors was proposed. This method used observations of a planar surface from different orientations. Their extended research [14] presents a calibration technique that uses perpendicular planes. Line segments were detected for the orthogonal planes and calibration was performed using the orthogonality and coplanarity of the line segments. Although these research studies may be extended to planes detected using 3D LiDAR sensors, it is expected that considerable modification of the implementation will be required.

An alternative to extrinsic calibration of LiDAR sensors is the use of artificial objects such as reflective tape, poles, or boxes. Underwood et al. [6] proposed a method for estimating the relative pose of a LiDAR sensor and a vehicle in which a vertical pole with reflective tape was used as a calibration object. Similarly, Gao et al. [7] placed several reflective tapes on vertical poles. The relative pose between LiDAR sensors and a vehicle were estimated by matching the reflective targets. In [8], a method for extrinsic calibration of a multi-LiDAR–multi-camera system using a hexahedron box was proposed. LiDAR–LiDAR calibration was performed by applying a singular value decomposition (SVD) to the vertices of the box.

Attempts have also been made to determine the extrinsic parameters using the quality of a 3D point cloud. Levinson and Thrun [15] proposed an unsupervised approach for intrinsic and extrinsic calibration of a high-resolution LiDAR sensor. An energy function was defined under the assumption that neighboring points lie on a contiguous surface. Intrinsic and extrinsic parameters minimizing the energy function were optimized using a grid search. Sheehan et al. [16] proposed a method for calibrating a rotating 3D LiDAR comprising three 2D LiDAR sensors. In their extended research, Maddern et al. [17] proposed an extrinsic calibration method of 2D and 3D LiDAR sensors. Rényi’s quadratic entropy (RQE) was used to measure the quality of a point cloud, while the extrinsic parameters were estimated by maximizing the quality function of the point cloud. However, these methods require an accurate trajectory of the vehicle.

Point cloud registration algorithms, such as iterative closest point (ICP) variants [1,2,3,4] and normal distribution transform (NDT) [5], can be exploited for the extrinsic calibration of 3D LiDAR sensors. In [18], the development of a LiDAR data set in urban environments is presented. To calibrate the extrinsic parameters between 3D LiDAR sensors, a generalized ICP [3] was applied to data from the overlapping region between the sensors. Trihedral features, such as the corner of a building, should exist in the overlapping regions. An open source software, Autoware [19], provided the implementation of various navigation algorithms for the autonomous vehicle. NDT was used for the extrinsic calibration of multiple LiDAR sensors. However, registration may fail if the point clouds are sparse or if the viewpoints of the sensors are significantly different. These studies, therefore, selected points for overlapping regions and applied them to the registration algorithms.

## 3. Problem Statement and System Overview

**Figure 1:**
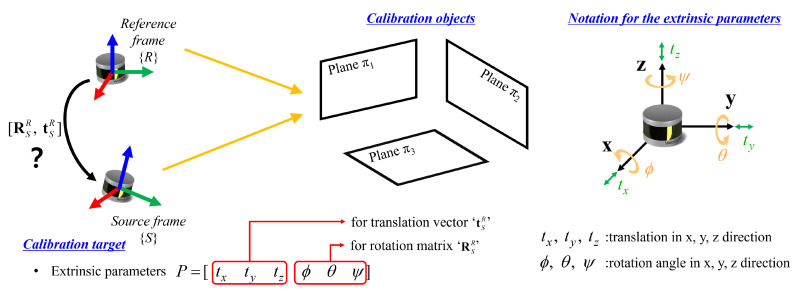
Problem statement for the proposed method. The aim of this research is to find extrinsic parameters that relate to the transformation between reference and source LiDAR sensors. The proposed method requires at least three non-parallel planes within the common field of view of the LiDAR sensors.

This paper describes a calibration method for extrinsic parameters of multiple LiDAR sensors mounted on a robotic or vehicular platform at different locations and directions. The extrinsic parameters refer to the relative position and the relative angle from the reference LiDAR sensors to the other sensors. The purpose of this study is to define the transformation between LiDAR sensors by accurately estimating the six degrees of freedom (DOF) extrinsic parameters $\left[t_{x},t_{y},t_{z},\phi ,\theta ,\psi \right]$, as shown in Figure 1.

There are two assumptions for the proposed method. The first one is that at least three non-parallel planes are required within the common field of view of the LiDAR sensors. It is necessary that the normal vector of the plane should span $R^{3}$ to estimate the 6DOF pose estimation of extrinsic parameters. The second is that the intrinsic parameter of each LiDAR sensor is assumed to be calibrated in advance. The intrinsic parameters relate to the internal optical components of the 3D LiDAR. If there is an error in the poses of the internal components, a distorted point cloud is generated, or a point cloud is entirely shifted from the origin of the LiDAR sensor. In this study, it is assumed that the intrinsic parameters are calibrated in a manufacturing process; consequently, there is neither distortion nor shift in the point clouds.

The initial poses of each LiDAR sensor can be defined by a human operator. We define the initial poses as modeling poses. The modeling pose refers to the user-defined extrinsic parameters between LiDAR sensors, which are mounted on a robot or a vehicle platform in different positions and orientations. It can be obtained via a drawing of the sensor mount or by measuring the relative distance and angles between sensors with a ruler and protractor. However, it is difficult to determine the modeling pose because not all detailed drawings of sensor mounts installed in a robotic or vehicular system are publicly available. Even if a detailed drawing is provided, installation errors or mechanical distortion of sensor mounts lead to inaccuracy in the modeling poses. Because the errors between the modeling poses and the actual poses are inevitable, this study aims to reduce these errors.

In this study, we set one of the sensors as a reference frame $\left\{R\right\}$ for calibration and calculate the relative poses between $\left\{R\right\}$ and remainder sensors. If the coordinate frames of the remainder sensors to be calibrated are defined as source frames $\left\{S\right\}$, the goal of this research is to determine the rotation matrix $R_{S}^{R}\in$ SO(3) and translation vector $t_{S}^{R}\in R^{3}$.

The proposed method is one approach to pairwise calibration [20,21]. However, calibration between source LiDAR sensors is not considered because the sensors have the same modality. It is sufficient to take one sensor as the reference and determine the transformation of the remainder of the LiDAR sensors in relation to it.

**Figure 2:**
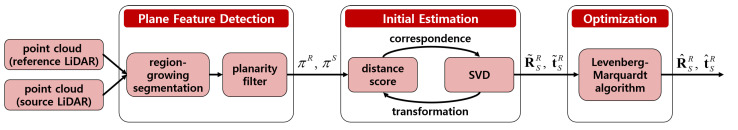
An outline of the proposed method. The calibration first performs a plane feature detection process for segmenting a point cloud and detecting plane objects. After that, an initial estimation of extrinsic parameters is performed through an iterative plane registration. The primary purpose of initial estimation is to find the right corresponding planes. Finally, the extrinsic parameters are refined through optimization using the measurement points within the corresponding planes.

Figure 2 presents an outline of the extrinsic calibration process. The transformation between $\left\{R\right\}$ and $\left\{S\right\}$ is calculated using plane features $\pi^{R}$ and $\pi^{S}$, which are detected by the reference and source LiDAR, respectively. The point cloud measured from each LiDAR sensor is segmented first, and plane objects to be used for calibration are detected by a planarity filter that considers the variance of points in the normal direction and the number of points in the segments. For sensors with different points of view, the initial estimation of the extrinsic parameters is performed by repeating the correspondence detection and matching the correspondence. The primary purpose of initial estimation is to find the right corresponding planes among plane objects measured by each sensor. An iterative plane registration process for finding and matching the corresponding planes is performed in the initial estimation. Instead of using the points, the plane parameters (normal, centroid, distance from the sensor origin) are used for plane registration. Subsequently, the extrinsic parameters are optimized by using the point clouds of the corresponding plane set. The optimization process aims to refine the extrinsic parameters obtained by initial estimation. The extrinsic parameters are jointly optimized to minimize the error between the measurement points and the plane.

## 4. Plane Feature Detection

In this section, we briefly describe the method of plane detection. First, segmentation of the point clouds is performed. There are some segmentation methods suitable for detecting plane features. A 3D LiDAR provides a dense point cloud in small-scale environments because the LiDAR is not far from the surrounding structures. In this case, we use region growing segmentation [22], which is based on the smoothness constraint of neighboring points. This method can divide the point cloud through smoothly connected areas. In contrast, in large-scale environments, a sufficiently dense point cloud for region growing segmentation cannot be obtained from low-resolution 3D LiDAR sensors. In this case, the dominant planes can be detected by MLESAC [23]. We use MLESAC for ground plane detection because low-resolution 3D LiDAR sensors do not provide enough ground points. For the segmentation of off-ground points, we used the region growing method.

A planarity filter is applied to the segmented point clouds to detect plane features. The filter tests three attributes concerned with the distribution of scan points on a segment. Let $x={\left(x_{i},y_{i},z_{i}\right)}^{T}$ be the scan point on the segment and $\bar{x}=\frac{1}{n}\sum_{i=1}^{n}x_{i}$ be the centroid of the segment. Then, the covariance of the segments is calculated using $C=\frac{1}{n-1}{\left(x-\bar{x}\right)}^{T}\left(x-\bar{x}\right)$. The eigenvectors of C are the principal components of the segments, and the eigenvalues are the explained variance of the principal components. The attributes of the segments are computed by using the sorted eigenvalues $\lambda_{1}\geq \lambda_{2}\geq \lambda_{3}\geq 0$.

The point cloud of a planar structure has eigenvalues $\lambda_{1}\approx \lambda_{2}\gg \lambda_{3}$. The first attribute of a segment is planarity $P_{\lambda }=\left(\lambda_{2}-\lambda_{3}\right)/\lambda_{1}$ as defined in [24,25]. The remaining attributes are explained variance $\lambda_{3}$ and the number of points (*n*). The segment to be considered as a plane feature should satisfy $P_{\lambda }\geq P_{\lambda ,thr}$, $\lambda_{3}\leq \lambda_{thr}$, and $n\geq N_{thr}$.

## 5. Extrinsic Calibration

### 5.1. Initial Transform Estimation through Plane Registration

The initial extrinsic parameters between 3D LiDAR sensors can be estimated by matching plane correspondences. The correspondence between two consecutive scans can easily be detected using the similarity of the plane parameters. However, if there are no given sensor poses or if the modeling pose errors are large, it is difficult to find plane correspondences at once. Furthermore, if the sensors have considerably different points of view, it is difficult to obtain a corresponding plane between the point clouds captured by each sensor. Because LiDAR sensors are mounted on a robot or a vehicle platform in different positions and orientations, even if the same wall is measured, other parts of the wall can be measured by each sensor. Moreover, the distance between each sensor and the plane object is also different. Therefore, the plane objects captured by each sensor can have different shapes, sizes, and poses. This situation is evident in a LiDAR sensor with low vertical resolution and a low vertical field of view. In essence, it is unknown which plane of the reference sensor corresponds to which plane of the source LiDAR sensor. The plane registration corresponds to lines 2–17 of Algorithm 1.


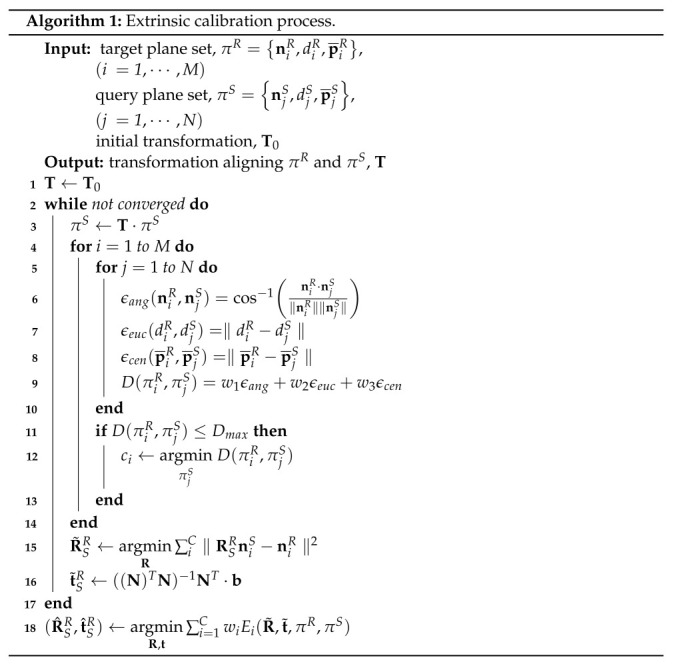


**Figure 3:**
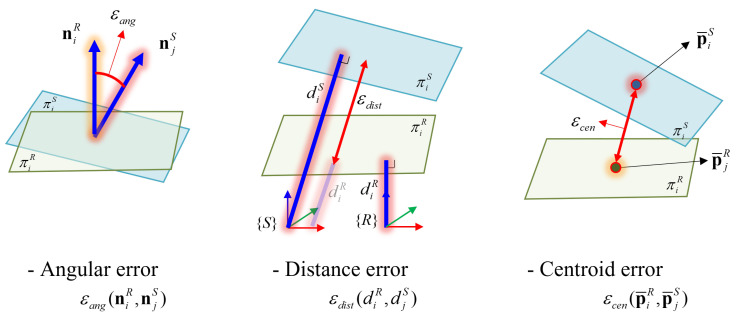
The test measures for assigning plane correspondences using the plane parameters.

The proposed method iterates the process of finding correspondences and matching the correspondences to determine the extrinsic parameters from unknown correspondences. Several test measures are calculated using the plane parameters to find the correspondences. Let $\pi^{R}$ and $\pi^{S}$ be planes detected by the reference and the source LiDAR sensor, respectively. Their plane parameters are $\left[{\left(n^{R}\right)}^{T},d^{R},\;{\left({\bar{p}}^{R}\right)}^{T}\right]$ and $\left[{\left(n^{S}\right)}^{T},d^{S},\;{\left({\bar{p}}^{S}\right)}^{T}\right]$, which consist of normal vector n, perpendicular distance *d* from the origin of each sensor, and centroid $\bar{p}$ of the measured plane. Figure 3 illustrates the test measures that are calculated from the plane parameters. The test measures are the differences between the planes in angle of normal vectors $\epsilon_{ang}$, distances $\epsilon_{euc}$, and centroids $\epsilon_{cen}$, which are computed based on lines 6–8 of Algorithm 1.

To detect corresponding planes, similarity distance $D(\pi_{i}^{R},\pi_{j}^{S})$ between the target and query plane, which is the weighted sum of the test measures, is calculated for all possible pairs of planes based on a brute-force search (line 9). *w* represents a weight for each test measure. Subsequently, the pair of planes with the smallest weighted sum is detected as plane correspondence (line 12). $c_{i}$ denotes the corresponding plane of the *i*-th target plane. If the similarity distance is above specific threshold value $D_{max}$, the pair of planes is rejected from the iteration of the calibration process.

To calculate the relative transformation between LiDAR sensors, the initial estimation of the extrinsic parameters is performed using the plane parameters of a set of plane correspondences. Let us define the correspondence planes of the reference sensor and the source sensor as $\pi_{i}^{R}$ and $\pi_{i}^{S}$(i=1,2,3,⋯,C), respectively. We also calculate the initial estimation of transformation $\left[{\tilde{R}}_{S}^{R},{\tilde{t}}_{S}^{R}\right]$. The geometric constraints of the corresponding planes to be matched are presented in the following equations:

(1) $$R_{S}^{R}n_{i}^{S}=n_{i}^{R}$$

(2) $$n_{i}^{R}\left(R_{S}^{R}{\bar{p}}_{i}^{S}+t_{S}^{R}\right)+d_{i}^{R}=0$$

Equation (1) is used to obtain rotation matrix $R_{S}^{R}$. As the planes have noises in their parameters, $R_{S}^{R}$ can be obtained via the least squares solution in line 15. A closed-form solution of line 15 can be obtained using the Kabsch algorithm [26]. Let us define the cross-covariance of a set of normal vectors of the corresponding planes as $H=\left[n_{1}^{S},\cdots ,n_{C}^{S}\right]{\left[n_{1}^{R},\cdots ,n_{C}^{R}\right]}^{T}$. If the singular value decomposition (SVD) of H is $U\Sigma V^{T}$, the rotation matrix is calculated using the following equation:

(3) $${\tilde{R}}_{S}^{R}=V\left(\begin{array}{ccc} 1 & 0 & 0\\ 0 & 1 & 0\\ 0 & 0 & det(VU^{T}) \end{array}\right)U^{T}$$

We can obtain a linear system by stacking (2) for the translation vector. The translation vector ${\tilde{t}}_{S}^{R}$ can be obtained from the following linear system:

(4) $$\begin{array}{cc} N{\tilde{t}}_{S}^{R} & =b\\ N=\left[\begin{array}{c} {\left(n_{1}^{R}\right)}^{T}\\ \vdots \\ {\left(n_{C}^{R}\right)}^{T} \end{array}\right],b & =\left[\begin{array}{c} -{\left(n_{1}^{R}\right)}^{T}R_{S}^{R}{\bar{p}}_{1}-d_{1}^{R}\\ \vdots \\ -{\left(n_{C}^{R}\right)}^{T}R_{S}^{R}{\bar{p}}_{C}-d_{C}^{R} \end{array}\right] \end{array}$$

The closed form solution of the least-squares problem in (4) is given by line 16.

In (3), the rank of the cross-covariance matrix H should be three in order to obtain a unique solution for a rotation matrix. Similarly, the set of normal vectors N in (4) should be invertible to obtain a unique solution. In essence, the rank of N should also be three. Consequently, at least three of the non-parallel planes are required to calculate the relative pose. The process for the plane registration is repeated until the sum of the similarity distance is converged. Therefore, The initial estimation is completed when the weighted sum $D_{k}=\sum_{i}^{C}D\left(\pi_{i}^{R},\pi_{i}^{S}\right)$ in the *k*-th iteration satisfies $D_{k-1}-D_{k}<\epsilon$.

### 5.2. Optimization of Calibration Parameters

In line 16 of Algorithm 1, the relative transformation between sensors is optimized using the scan points on the planes. A set of parameters $\Theta =[t_{x},t_{y},t_{z},\phi ,\theta ,\psi ]$ that includes Euler angles, and a translation vector is jointly estimated in the optimization process. The projection of the points on a source plane onto its plane correspondence should be minimized. Optimized transformation ${\hat{R}}_{S}^{R}$ and ${\hat{t}}_{S}^{R}$ can be obtained by minimizing the following cost function:

(5) $$\begin{array}{cc} \left({\hat{R}}_{S}^{R},{\hat{t}}_{S}^{R}\right) & \leftarrow \underset{R,t}{\mathrm{argmin}}\sum_{i=1}^{C}w_{i}E_{i}\left(\tilde{R},\tilde{t},\pi^{R},\pi^{S}\right)\\ E_{i}\left(\tilde{R},\tilde{t},\pi_{i}^{R},\pi_{i}^{S}\right) & =\sum_{p\in \pi^{s}}{\|n_{i}^{R}\cdot \left({\tilde{R}}_{S}^{R}p^{S}+{\tilde{t}}_{S}^{R}\right)+d_{i}^{R}\|}^{2}, \end{array}$$

where *C* is the number of plane correspondences, and $\omega_{i}$ is the weight reflecting the uncertainty of the *i*-th plane correspondence.

The weight of the correspondence is computed as the inverse of the variance along the normal direction: $\omega_{i}=1/{\left(\sigma_{i}^{S}\right)}^{2}$. In this study, we applied the Levenberg–Marquardt (LM) algorithm [27] to solve a non-linear least square problem. The purpose of the LM method is to iteratively estimate calibration parameters Θ that minimize the cost function. Let us define the residual satisfying $E_{i}\left(\tilde{R},\tilde{t},\pi_{i}^{R},\pi_{i}^{S}\right)=\sum_{p\in \pi^{s}}{\left\|e_{i}\left(\Theta \right)\right\|}^{2}$ as $e_{i}\left(\Theta \right)$. The Jacobian matrix for the parameter estimation is given as follows:

(6) $$J_{i}=\frac{\partial e_{i}}{\partial \Theta },\;\;e_{i}=\frac{1}{\sigma_{i}^{S}}\left(n_{i}^{R}\left(R_{S}^{R}p^{S}+t_{S}^{R}\right)+d_{i}^{R}\right)$$

The length of Jacobian matrix J is $\sum_{i=1}^{M}N_{i}$, and the dimension is six, which is the same as that of the set of parameters. We set the transformation obtained from Section 5.1 as the initial values of the optimization process, and the updated parameters in each LM iteration are given by the following equation:

(7) $$\Theta_{k+1}=\Theta_{k}-{\left(J^{T}J+\lambda diag\left(J^{T}J\right)\right)}^{-1}\left(J^{T}e\right)$$

## 6. Experiments

### 6.1. Experimental Setup

**Figure 4:**
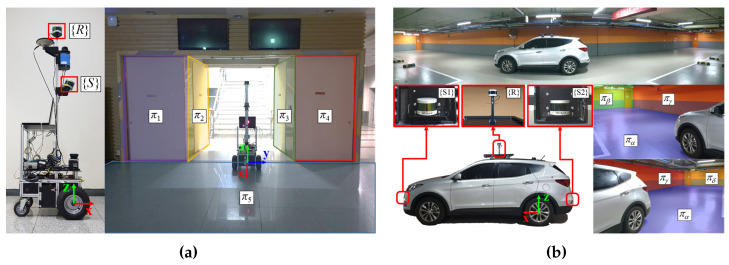
Experimental setup (**a**) a robotic system (**b**) a vehicular system.

To validate our calibration approach, we conducted experiments using a robotic and a vehicular system. The systems were equipped with multiple low-resolution 3D LiDAR sensors (Velodyne VLP-16). Two LiDAR sensors were mounted on the robotic platform as shown in Figure 4a. One was installed horizontally at a height of 1.9 m, and the other was tilted about 22.5${}^{\circ }$ toward the ground at a height of 1.4 m. We set the horizontally mounted LiDAR sensor as the reference frame $\left\{R\right\}$, and the tilted LiDAR sensor was considered as the source frame $\left\{S\right\}$. The calibration of the relative transformation between $\left\{R\right\}$ and $\left\{S\right\}$ was performed in the environment shown in the right side of Figure 4a.

Figure 4b shows the vehicular system and the experimental environment. Similar to the robotics system, the vehicle was equipped with three LiDAR sensors to minimize blind spots. A sensor was attached to the top, front, and rear of the vehicle. We set the sensor on the top of the vehicle as the reference frame $\left\{R\right\}$. The front and rear sensors were set as source frames $\left\{S1\right\}$ and $\left\{S2\right\}$, respectively. The extrinsic calibration of the vehicular system was performed in an underground parking lot. The plane objects on the front and the rear side of the vehicle were used as calibration objects.

### 6.2. Extrinsic Calibration of Robotics System

**Figure 5:**
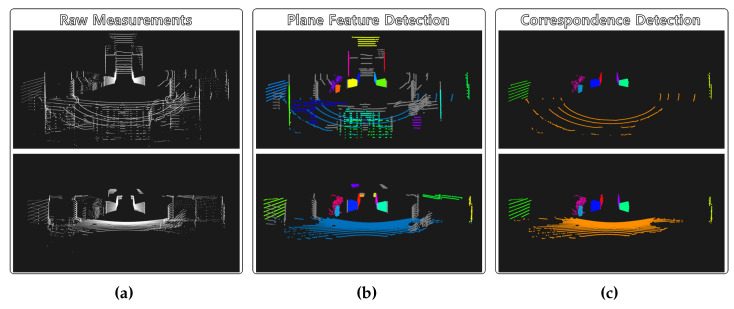
Process of plane correspondence detection. Each row corresponds to the reference and source LiDAR sensors. Each column corresponds to (**a**) raw measurements, (**b**) plane object detection, and (**c**) correspondence detection.

For the first step of the extrinsic calibration, we detected the plane features in the target environment and found the correspondences. Figure 5 shows the process of plane correspondence detection. Figure 5a shows the raw point cloud collected from each LiDAR sensor. The ground plane was extracted using MLESAC on account of a sparse point cloud on the ground. Then, we detected other plane features using region growing segmentation. For the segmentation of the off-ground point cloud, we computed the normal vector of each scan point using its neighboring points. In this study, we set the number of the neighboring points to calculate the point normal to 30. Then, the 30 nearest neighbors of the current seed point were used to group the points belonging to the smooth surface. We set the thresholds for the smoothness angle and the residual to 10.0${}^{\circ }$ and 10.0 $m^{-1}$.

The planarity filter stated in Section 4 was applied to the segmented point clouds. Figure 5b shows the plane features detected by using the planarity filters. The upper figure is the result from the reference sensor, and the lower figure is the result from the source sensor. The colored points represent segments that correspond to the detected planes, and parts other than the plane are expressed in gray.

Because we did not know the correspondences between the planes detected by each sensor, the iterative process of finding correspondences and matching them was performed to detect the plane correspondence and to simultaneously compute the initial transform $\left[{\tilde{R}}_{S}^{R},{\tilde{t}}_{S}^{R}\right]$. Figure 5c shows the result of deriving the correspondences from the plane segments in Figure 5b. Each plane correspondence is expressed in the same color. There were a total of nine plane correspondences in the target environment.

**Figure 6:**
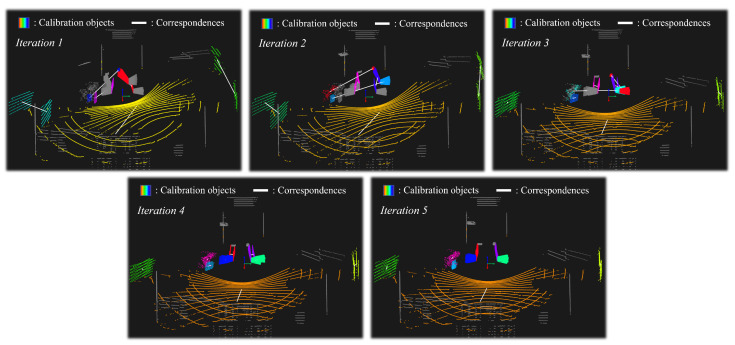
Process of plane registration in a challenging scenario where the initial modeling pose is significantly different from the ground truth. Each pair of corresponding planes is marked with the same color and connected by a white line.

Figure 6 shows a process of plane registration in a challenging scenario where the initial modeling pose is significantly different from the ground truth. The shape of the point cloud with respect to the ground (orange color) is different for each LiDAR sensor. This is because the reference LiDAR is mounted facing forward, while the source LiDAR is tilted toward the ground. Different plane shapes and errors in modeling poses make it challenging to find a corresponding plane. In this scenario, the point clouds are registered while finding the correct corresponding plane after several iterations by the proposed plane matching algorithm.

**Figure 7:**
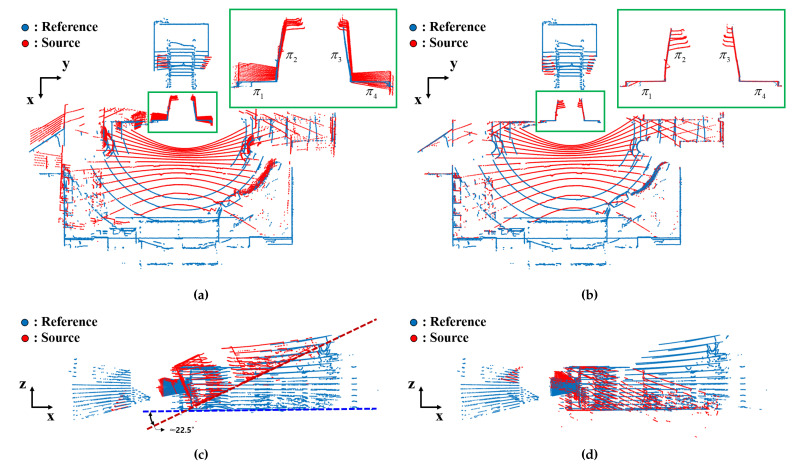
Point clouds from the reference (blue dots) and source (red dots) LiDAR sensors of the robotic system. Top view of the point cloud (**a**) before and (**b**) after calibration. The magnified area of the top views represent the environment shown in Figure 4a. Side view of the point cloud (**c**) before and (**d**) after calibration. The dashed lines in the side views correspond to the ground planes.

Figure 7a,c show the raw point clouds of the target environment before the extrinsic calibration. The blue points represent the point cloud measured by the reference LiDAR sensor, and the red points are from the tilted LiDAR sensor. The green bounding box in Figure 7a corresponds to the environment in Figure 4a. If the transformation between sensors was correct, the blue and red points for plane $\pi_{1}∼\pi_{4}$ should be matched. However, it can be seen that the two point clouds are not aligned. Figure 7c shows the side view of the point clouds. The ground plane in each point cloud is expressed as a dashed line. It can be seen that the point cloud from the tilted LiDAR sensor is inclined by about 22${}^{\circ }$ compared to the point cloud of the reference sensor.

Optimized transform $\left[{\hat{R}}_{S}^{R},{\hat{t}}_{S}^{R}\right]$ is calculated through the LM iteration. The calibration parameters obtained from the initial estimation process were set as the initial values of the optimization process. The resultant transformation was applied to the point cloud of the tilted sensor. The results of the extrinsic calibration are shown in Figure 7b,d, which are the top view and the side view of the point cloud, respectively. In the magnified view of Figure 7b, the blue and the red point clouds that correspond to plane $\pi_{1}∼\pi_{4}$ were perfectly matched. Furthermore, the ground surfaces were also aligned with each other, as shown in Figure 7d. Therefore, it was verified that the extrinsic parameters between the two LiDAR sensors were successfully calibrated using the proposed method.

### 6.3. Extrinsic Calibration of Vehicular System

**Figure 8:**
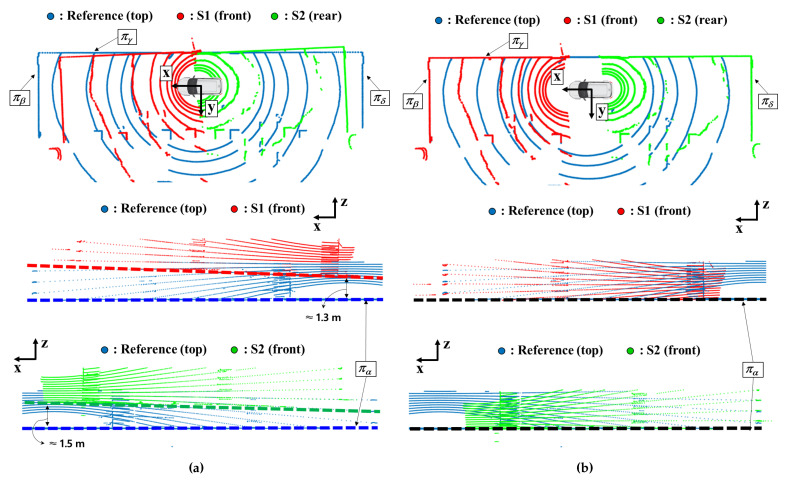
Point clouds from the reference (blue dots), front (red dots), and rear (green dots) LiDAR sensors of the vehicular system. (**a**) the point clouds before calibration. (**b**) the point clouds after calibration. Each row of the figures corresponds to the top view (**upper**), side view of the front (**middle**), and side view of the rear (**lower**), respectively. The dashed lines in the side views correspond to the ground planes.

The modeling poses of the LiDAR sensors were not set except for a 180-degree rotation on the *z*-axis for the rear sensor. The point clouds before calibration are shown in Figure 8a. The blue, red, and green points represent the point clouds acquired from the top, front, and rear sensors. The upper panel of Figure 8a shows a top view of the point clouds. It was observed that the discrepancies in point clouds were evident on plane $\pi_{\beta }∼\pi_{\delta }$. The front and rear point clouds were rotated on the z-axis about 2.5${}^{\circ }$. The middle and lower panels of Figure 8a show side views of the point clouds. The dashed lines represent the ground planes, which correspond to plane $\pi_{\alpha }$ in Figure 4b. At the center of the reference sensor, the distance between the ground planes of the reference and front sensors was approximately 1.3 m, and the angle difference was about 2.2${}^{\circ }$. Similarly, for the ground plane of the rear sensor, the differences from the ground plane of the reference sensor were 1.5 m in height and 2.5${}^{\circ }$ in angle.

Extrinsic calibration of the vehicular system was also performed using the proposed method. The entire process was the same as the calibration process for the robotic system. For the process of detecting plane features, the parameters applied to the segmentation methods and planarity filter were the same as those for the robotic system. The point clouds transformed by the determined extrinsic parameters are shown in Figure 8b. In the top view, the point clouds that correspond to side wall $\pi_{\beta }∼\pi_{\delta }$ were perfectly aligned. Furthermore, the ground planes were also matched with each other, as shown in the middle and lower panels in Figure 8b. Therefore, it was verified that the extrinsic parameters of the LiDAR sensors were successfully calibrated using the proposed method.

### 6.4. Evaluation

To evaluate our calibration approach, we compared the proposed method with ICP [1] and its variants [2,3,4] and NDT [5]. One of the contributions of this paper is the plane registration technique, which can easily automate the entire calibration process without human intervention. The conventional work for plane-based extrinsic calibration requires a process in which a user manually assigns corresponding planes for a plane pair [28]. Therefore, it was not included in the comparison. There are other conventional works that automatically calibrate extrinsic parameters using GICP [18] and NDT [19]. GICP uses plane-to-plane features using point normal information for matching. The four types of ICP variants considered for the evaluation were point-to-point ICP (pt-ICP) [1], point-to-plane ICP (pl-ICP) [2], generalized-ICP (GICP) [3,18], and normal ICP (NICP) [4]. Since NICP uses the normal, curvature, and projection distance of a local plane, it can be seen as a method using plane features. When a raw point cloud was applied to the registration algorithms, alignment often failed due to the cluttering of the points. In this study, the point clouds of the plane segments detected in Section 4 were applied to the registration algorithms. The point cloud of each sensor was downsampled using a grid size of 0.1 m. The NDT grid size was set to 2.0 m.

Table 1 shows the results for the quantitative evaluation. The quantitative measure for the evaluation was the root mean square error (RMSE) between the plane model and the scan points for all plane correspondences.

The RMSE for the *k*-th corresponding plane pair is calculated as follows.

(8) $${\mathrm{RMSE}}_{k}=\sqrt{\frac{1}{N}\sum_{i=1}^{N}{\left(\frac{n_{k}^{R}p_{i}+d_{k}^{R}}{\left|\right|n_{k}^{R}\left|\right|}\right)}^{2}},$$

where $n_{k}^{R}$ is the normal vector of the reference plane, and $d_{k}^{R}$ is the distance from the origin to the reference plane. $p_{i}$ is the *i*-th measurement point belonging to the *k*-th corresponding plane pair, and *N* is the total number of measurement points of a pair of correspondence planes.

For all methods, the RMSE was calculated for three cases: the ground plane, off-ground planes, and all planes. The lower value of the RMSE represents a more accurate extrinsic calibration result.

The evaluation result on the robotic system is shown in the row referred to as (a) in Table 1. The proposed method showed the lowest RMSE values for the three cases. In particular, the RMSE value for the ground showed a noticeable difference compared to other registration methods, except for GICP. The RMSE value of all planes measured by the reference LiDAR was about 0.0153 m. The proposed method showed results similar to the planes measured using a single sensor. Among the conventional methods, the RMSE values of the GICP were the closest to that of the proposed method. However, the proposed method showed a slightly better performance. Although pt-ICP and 3D NDT showed RMSE values for the off-ground close to the values of the proposed method, the RMSE values for the ground plane were more than twice those of the proposed method. These results appear because the registration accuracy is low due to the insufficient number of corresponding points on the ground plane. Consequently, the RMSE values for all planes were also twice as large compared to those for the proposed method. The RMSE values of pl-ICP were more than three times compared to those for the proposed method in all cases.

The evaluation results on the vehicular system are shown in the rows referred to as (b) and (c) in Table 1. The proposed method also showed the lowest RMSE values for all scenarios. The RMSE value of all planes that were measured by the reference LiDAR is about 0.0254 m. As with the robot system, the overall RMSE values from the proposed method is 0.0290 m, which is close to the value of the reference sensor. The GICP showed better performance than other registration methods. However, in $\left\{R\right\}$ to $\left\{S2\right\}$ calibration, the RMSE value for the ground is 0.0761 m, which is four times greater than that of the proposed method. This is because there were few overlaps between the reference sensor and the rear sensor in the ground data. The ground RMSE values of pt-ICP, pl-ICP, and NICP are above 0.1 m, which implies that they practically failed in registration.

NICP and NDT failed to match the two sets of point clouds from the vehicular system when the initial translation was not given. We additionally set up the initial translation of the source LiDAR sensors. The initial translation was set to 2 m for $\left\{S1\right\}$ LiDAR and -2 m for $\left\{S2\right\}$ LiDAR in the direction of the *x*-axis of the reference coordinate system. Although the initial translation was given, the RMSE values for the ground planes were found to be more than 0.2 m.

The conventional registration methods shared a lower accuracy for the ground plane matching. The reason for this result is that the methods require a large number of corresponding points in overlapping regions. In the experiments, VLP-16 provides a sparse point cloud in a single scan. Furthermore, there were only small overlapping regions on the ground surfaces because the points of view of the LiDAR sensors were quite different. Therefore, the number of point correspondences on the ground was smaller than those not on the ground. Consequently, ICP variants and NDT did not achieve convergence on the ground planes. However, the proposed method was robust enough for the sensor configuration, where the points of view of the sensors were quite different because it utilized the plane models and variances of the plane correspondences.

## 7. Conclusions

In this paper, we have presented a method for extrinsic calibration of multiple 3D LiDAR sensors using planar features. The only requirement is more than three independent planes that can easily be found in surrounding environments. Therefore, the proposed method does not require any environmental modifications. A plane registration method is presented to deal with cases in which the viewpoints of the LiDARs are significantly different. The proposed method iterates the process of assigning correspondences and aligning the correspondences to estimate the initial transformation. The uncertainties of the plane are also considered in the optimization process to lower the effect of the noisy plane.

We conducted several experiments on a robotic and a vehicular system to validate our calibration algorithm. For evaluation, we compared the proposed method with ICP variants and 3D NDT. The proposed method showed better performance than the conventional registration methods in terms of accuracy. Therefore, we have demonstrated that the proposed method achieves robust performance using sensors with different points of view.

## Figures and Tables

**Table 1:** Root mean square errors of the plane correspondences. (a) A robotic system, (b) vehicular system (*R* to S1), (c) vehicular system (*R* to S2).

Scene	(Unit: m)	Proposed	pt-ICP	pl-ICP	GICP	NICP	NDT
(a)	ground	**0.0144**	0.0497	0.0564	0.0161	0.0358	0.0337
	non-ground	**0.0175**	0.0200	0.0587	**0.0175**	0.0415	**0.0175**
	overall	**0.0159**	0.0391	0.0574	0.0167	0.0385	0.0275
(b)	ground	**0.0150**	0.2341	0.1015	0.0181	0.2377	0.3328
	non-ground	**0.0381**	0.0853	0.0512	0.0397	0.1168	0.0564
	overall	**0.0290**	0.1766	0.0806	0.0308	0.1876	0.2394
(c)	ground	**0.0173**	0.3308	0.1910	0.0761	0.6734	0.6435
	non-ground	**0.0362**	0.0570	0.0400	0.0371	0.1433	0.0527
	overall	**0.0286**	0.2344	0.1363	0.0594	0.4748	0.4453

## Data Availability

Not applicable.
